# Affordable artificial intelligence-based digital pathology for neglected tropical diseases: A proof-of-concept for the detection of soil-transmitted helminths and *Schistosoma mansoni* eggs in Kato-Katz stool thick smears

**DOI:** 10.1371/journal.pntd.0010500

**Published:** 2022-06-17

**Authors:** Peter Ward, Peter Dahlberg, Ole Lagatie, Joel Larsson, August Tynong, Johnny Vlaminck, Matthias Zumpe, Shaali Ame, Mio Ayana, Virak Khieu, Zeleke Mekonnen, Maurice Odiere, Tsegaye Yohannes, Sofie Van Hoecke, Bruno Levecke, Lieven J. Stuyver

**Affiliations:** 1 Etteplan Sweden AB, Uppsala, Sweden; 2 Janssen Global Public Health, Janssen R&D, Beerse, Belgium; 3 Department of Translational Physiology, Infectiology and Public Health, Ghent University, Merelbeke, Belgium; 4 Laboratory Division, Public Health Laboratory-Ivo de Carneri, Chake Chake, United Republic Tanzania; 5 Jimma University Institute of Health, Jimma, Ethiopia; 6 National Centre for Parasitology, Entomology and Malarial Control, Ministry of Health, Phnom Penh, Cambodia; 7 Kenya Medical Research Institute, Kisumu, Kenya; 8 Arba Minch University, Arba Minch, Ethiopia; 9 IDLab, Department of Electronics and Information Systems, University of Ghent—Imec, Zwijnaarde, Belgium; NIPD: National Institute of Parasitic Diseases, CHINA

## Abstract

**Background:**

With the World Health Organization’s (WHO) publication of the 2021–2030 neglected tropical diseases (NTDs) roadmap, the current gap in global diagnostics became painfully apparent. Improving existing diagnostic standards with state-of-the-art technology and artificial intelligence has the potential to close this gap.

**Methodology/Principal findings:**

We prototyped an artificial intelligence-based digital pathology (AI-DP) device to explore automated scanning and detection of helminth eggs in stool prepared with the Kato-Katz (KK) technique, the current diagnostic standard for diagnosing soil-transmitted helminths (STHs; *Ascaris lumbricoides*, *Trichuris trichiura* and hookworms) and *Schistosoma mansoni* (SCH) infections. First, we embedded a prototype whole slide imaging scanner into field studies in Cambodia, Ethiopia, Kenya and Tanzania. With the scanner, over 300 KK stool thick smears were scanned, resulting in total of 7,780 field-of-view (FOV) images containing 16,990 annotated helminth eggs (*Ascaris*: 8,600; *Trichuris*: 4,083; hookworms: 3,623; SCH: 684). Around 90% of the annotated eggs were used to train a deep learning-based object detection model. From an unseen test set of 752 FOV images containing 1,671 manually verified STH and SCH eggs (the remaining 10% of annotated eggs), our trained object detection model extracted and classified helminth eggs from co-infected FOV images in KK stool thick smears, achieving a weighted average precision (± standard deviation) of 94.9% ± 0.8% and a weighted average recall of 96.1% ± 2.1% across all four helminth egg species.

**Conclusions/Significance:**

We present a proof-of-concept for an AI-DP device for automated scanning and detection of helminth eggs in KK stool thick smears. We identified obstacles that need to be addressed before the diagnostic performance can be evaluated against the target product profiles for both STH and SCH. Given that these obstacles are primarily associated with the required hardware and scanning methodology, opposed to the feasibility of AI-based results, we are hopeful that this research can support the 2030 NTDs road map and eventually other poverty-related diseases for which microscopy is the diagnostic standard.

## Author summary

Recently, the World Health Organization (WHO) published its 2021–2030 road map for neglected tropical diseases (NTDs). While diagnostics are clearly pivotal to steer the NTD endemic countries towards the ambitious targets set, the current gap in the global diagnostic armamentarium for NTDs once more becomes painfully apparent. As most NTD programs mainly rely on microscopic examination of slides, automation of slide scanning coupled with artificial intelligence (AI) has the potential to close this diagnostic gap by 2030. Therefore, we developed a device to automate scanning of stool smears and deployed it in four endemic countries to build an image data base for both intestinal and blood-dwelling worms. After the images were annotated, we used 90% to train and 10% to validate an AI model. As the AI model was able to reliably recognise worm eggs, we provided a proof-of-concept for automated scanning and detection of worm eggs in stool smears. We identified important obstacles for both the slide scanning device and the application of AI, but we are hopeful that this research can support the 2030 NTDs roadmap and eventually other NTDs for which microscopic examination is the diagnostic standard.

## Introduction

Recently, the World Health Organization (WHO) published its 2021–2030 road map for neglected tropical diseases (NTDs) [1]. The four overarching global targets are (i) a 90% reduction in people requiring interventions against NTDs, (ii) a 75% reduction in disability-adjusted life years attributable to NTDs, (iii) elimination of at least one NTD in 100 countries, and (iv) eradication of two NTDs [1]. While it is clear that diagnostics are pivotal to steer the NTD endemic countries towards the ambitious targets set, the current gap in the global diagnostic armamentarium for NTDs once more becomes painfully apparent [1,2]. Indeed, a variety of NTD control programs still rely heavily on the microscopic examination of specimens (blood: lymphatic filariasis; urine: urinary schistosomiasis; stool: intestinal schistosomiasis; soil-transmitted helminthiasis; foodborne trematodiasis: clonorchiasis, fascioliasis, opisthorchiasis and paragonimiasis, and taeniasis; skin: onchocerciasis). While microscopic examination is often a cheap and simple procedure, it has some critical shortcomings including but not limited to poor sensitivity and reproducibility, and an error-prone manual read-out [3].

As a response to this lack of diagnostics armamentarium, the WHO has established a dedicated working group (Diagnostics and Technical Advisory Group (DTAG)) to identify and prioritise diagnostic needs, and to subsequently develop target product profiles (TPPs) for future diagnostics [4,5]. These TPPs describe the minimal and ideal requirements for various diagnostic needs (e.g., simplicity, performance, and price of the test) related to NTD specific use-cases (start, monitor and evaluate program performance *vs*. verify whether transmission of diseases has been interrupted). Today, the WHO already published TPPs for lymphatic filariasis [6], onchocerciasis [7], soil-transmitted helminthiasis [8], and schistosomiasis [9], while TPPs for other NTDs are in preparation. However, the pathway to translate new diagnostic biomarkers from initial discovery into an affordable laboratory or point-of-care diagnostic test for endemic programs remains long, expensive, and uncertain [10,11]. As a consequence of this, prioritising diagnostics based on new biomarkers for the WHO targets is extremely ambitious, and in the long run, may turn into an “appeal-to-future-discovery fallacy” [3].

An alternative diagnostic approach to these new biomarkers-based platforms, is to build upon the widely accepted microscopic procedures by implementing artificial intelligence-based digital pathology (AI-DP). AI-DP is a revolutionising concept in general clinical pathology and may also contribute significantly to NTD programs. The application of AI-DP may overcome the critical shortcomings of current microscopic diagnostic standards, such as poor reproducibility and error-prone manual read-out [3]. Furthermore, a digitalised workflow can create opportunities to reduce operational costs (by increasing throughput) and automate data capture, analysis and reporting [3]. An AI-DP solution for field settings may consist of a whole slide imaging (WSI) scanner, an AI model, and a data reporting system. But as of today, the integration of AI-DP technology applications still faces several challenges. The primary constraints for implementation are (i) affordability: current WSI scanners remain expensive, (ii) performance: achieving a sample throughput, clinical sensitivity and clinical specificity comparable/superior to the current diagnostic standards remains challenging, (iii) operability: it is difficult to achieve reliable operation of the diagnostic tools in endemic communities with limited infrastructure, and (iv) usability: the solution should be simple and comparable/superior to the current standard methods.

We present a proof-of-concept for the detection of soil-transmitted helminth (STH) and intestinal *Schistosoma* (*Schistosoma mansoni* (SCH)) eggs in Kato-Katz (KK) stool thick smears using an affordable AI-DP prototype configuration. To this end, we engineered an early prototype of a WSI scanner and used it in field studies in Cambodia, Ethiopia, Kenya and Tanzania. Scanning KK stool thick smears allowed us to build an image database of STH and SCH eggs. Eggs were subsequently annotated, with approximately 90% being used to train and 10% being used to evaluate the performance of our artificial neural network-based object detection model for each of the helminths. The combination of field testing with a purpose-built prototype WSI scanner and deep learning approach allowed us to critically assess the obstacles for introducing AI-DP to support monitoring and evaluation of control programs designed to reduce the morbidity attributed to STH and SCH.

## Methods

### Ethics statement

Two field trials were embedded into the Stop Anthelminthic Resistant Worms (Starworms; https://www.starworms.org/) project (Cambodia and Tanzania), one through the Morbidity Operational Research for Bilharziasis Implementation Decisions (MORBID, https://www.ntdsupport.org/cor-ntd/ntd-connector/morbid-morbidity-operational-research-bilharziasis-implementation-decisions) project (Kenya) and one clinical study to assess the effect of intermittent iron and vitamin A supplement on nutritional status and development of school children (Ethiopia). With exception of the MORBID project, all study protocols were approved by the institutional review board of the University Hospital and the Faculty of Medicine and Health Sciences of Ghent University, Belgium. The study protocols were also approved by respective institutional review boards (Cambodia: EC2018/1038, Ministry of Health of Cambodia (NEHCR 101); Ethiopia: EC2019/1289, Arba Minch University; Pemba Island (Tanzania): EC2018/1141, Zanzibar Health Research Institute (Tanzania, ZAHREC/03/DEC/2018); and Kenya: NTD-SC/NCT #:158, Safe Water and AIDS Project (SWAP), Kisumu). Formal written consent was obtained from parent/guardian.

No personal data of study participants have been stored in our database. Targeting different geographical areas allowed us to image the desired STH and SCH species and capture variations in stool colour and consistency. High infection stool samples were targeted to maximise the number of imaged eggs while minimising the total number of KK stool thick smears to be prepared and scanned.

**Fig 1:**
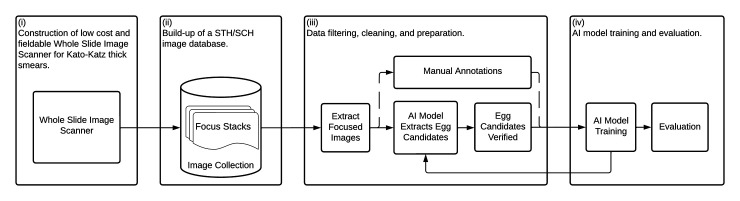
The process used to explore the feasibility of an artificial-intelligence-based-digital-pathology device.

To demonstrate the feasibility of an AI-DP device for STHs and SCH, we focused on four activities: (i) design of the WSI scanner, (ii) build-up of the image database, (iii) annotation of the images, and (iv) AI training and evaluation (**Fig 1**).

### Design of the whole slide image scanner

**Fig 2:**
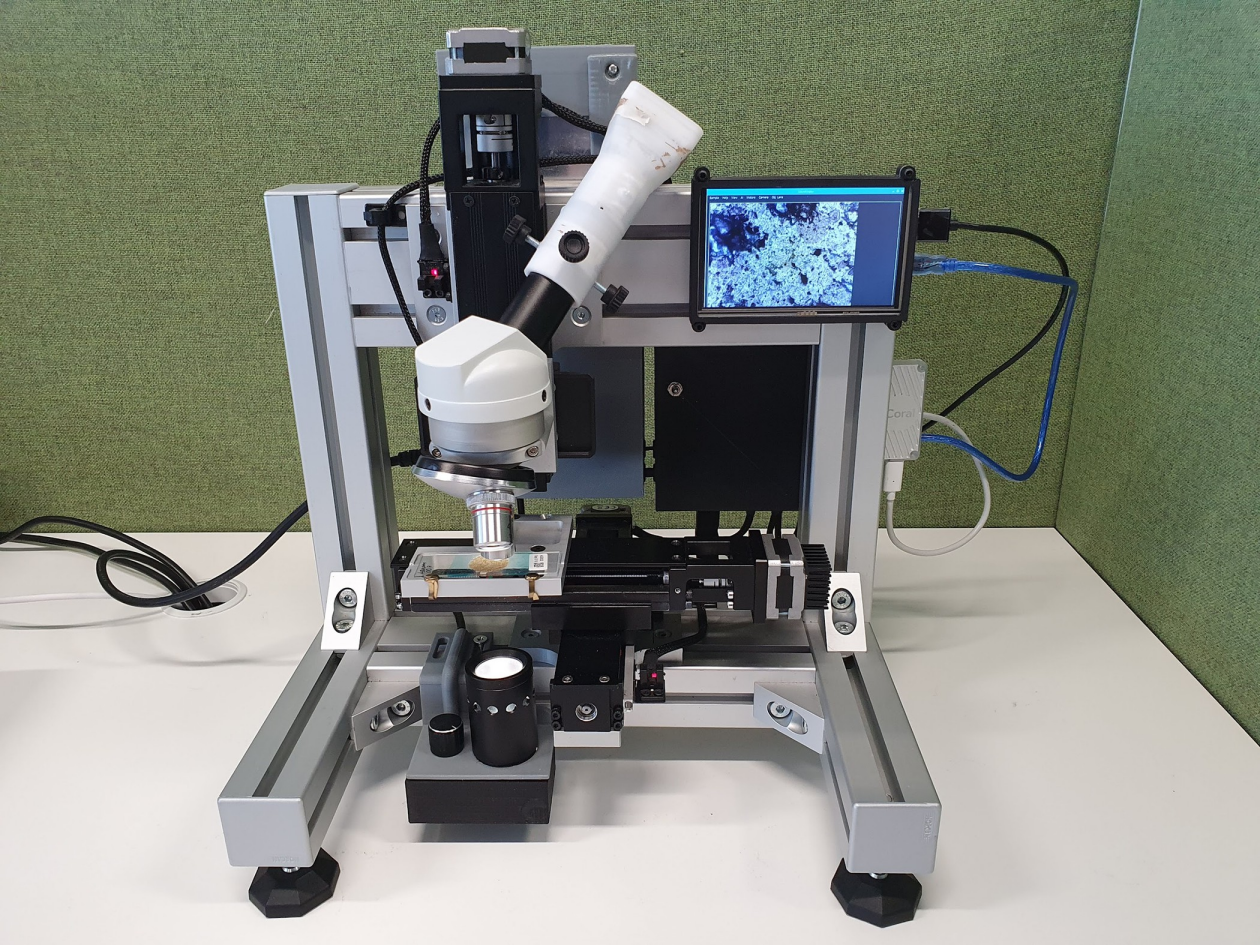
A prototype of a low-cost fieldable whole slide imaging device for Kato-Katz stool thick smears.

Core to the AI-DP solution for STH and SCH is a WSI scanner capable of capturing focused digital images for every field-of-view (FOV) within a sample. However, no commercial solutions were identified to achieve the TPP requirements outlined by the WHO when considering performance, usability, portability, operability, and a price target within USD 5,000. Although several research groups have been working on low-cost, automated and fieldable WSI scanners [12–19], they have not demonstrated the ability to automatically scan complete stool thick smears with reasonable throughput, and are not readily available for use in the field. Furthermore, we realised that the success of developing a deep learning-based AI model lies in creating an expansive database of STH and SCH eggs in human stool samples prepared according to the KK technique in real-world study settings. We therefore designed and engineered our own fieldable WSI scanner (**Fig 2**), focusing on low-cost, modular, and off-the-shelf components that offered the flexibility to explore different scan configurations, optics configurations, magnifications, image sensors and lighting solutions. As an exploratory prototype, we did not set out a complete development plan to meet the TPP requirements outlined by WHO; instead, we focused on flexibility and high-quality data capture. At under USD 2,000, the bill of materials for the WSI scanner is given in **S1 Info**.

### Build-up of the image database

#### Origin of the images

The WSI scanner was taken into the field to collect images from KK stool thick smears slides that were prepared as part of ongoing studies run in Cambodia (Krong Kampong Chhnang), Ethiopia (Arba Minch), Tanzania (Pemba Island), and Kenya (Kisumu).

#### Annotation of the images

The annotation of images occurred in six consecutive steps. In the **first step**, a non-exhaustive set of focus stacks containing STH and SCH eggs were identified from all collected images. The most in-focus FOV image for each focus stack was recorded by visual confirmation. In the **second step**, we manually annotated the focused images with both the location and the identity of each helminth egg (*Ascaris*, *Trichuris*, hookworm, or SCH). More specifically, we manually annotated the FOV images by drawing rectangular boxes around helminth eggs. For this, we used LabelImg, an open-source graphical image annotation tool [20]. The goal was to annotate approximately 100 eggs for each STH and SCH helminth species while targeting different stool samples to provide a wide variation in the appearance of images. In the **third step**, we trained an object detection model (see next section) to automatically extract potential helminth eggs from the remaining images. The new egg candidates were presented to experienced users in a purpose-built graphical user interface (GUI) for manual verification and further model training. In the **fourth step**, we applied the trained model to the remaining focused FOV images to find any eggs that may have been missed in the previous step. Again, the potential helminth eggs were verified by users with the GUI. This iterative approach (repeating the third and fourth step) considerably simplified the extraction process (and annotation of eggs from FOV images) but did not guarantee that all helminth eggs were extracted from the FOV images. Therefore, we used LabelImg again to review all focused FOV images in an unseen ground truth test set (extracting the test set is explained in next section) for any missed helminth eggs in the **sixth step**. This final step ensures accurate evaluation of the final AI model against our unseen ground truth test set.

#### AI training and evaluation

Once the confirmed helminth eggs were extracted and verified, FOV images were randomly shuffled and split into a three data sets, namely a training set, a validation set and a testing set. When constructing these data sets of FOV images, we aimed for a desired split ratio of 70:20:10 for each helminth eggs species in each data set. As the FOV images contained multiple eggs and may include mixed infections it was not possible to precisely split into the target percentages. The training and validation sets were used during the training process to develop the model. The test set was used as an unseen set of data withheld from the training process and was used only for the final evaluation of the models.

Using the annotated training set, a convolutional neural network (CNN) was trained. While there are numerous types and combinations of neural network architectures that can be used for image classification and object detection, most state-of-the-art models are based on CNN [21] as they can capture spatial dependencies (one pixel value is often dependent on its neighbouring pixels) through the use kernels or filters, which are convolved over the image to reduce dimensionality and extract features from the image. The extracted features depend on the size of the filter and elements within the filter. For example, a 3x3 filter may be constructed with elements that perform edge detection of vertical lines. However, rather than manually defining the properties of the filters (as is done in traditional computer vision approaches), the properties can be optimised in the training process. CNN-based architectures vary in approach by the number of layers used, the number of filters per layer and the size of filters. When considering the purpose of object detection, there are typically two steps, namely finding the objects in the image and classifying the objects. While some approaches may adopt a single stage CNN approach, others may add additional steps or techniques to separate the problem [22]. Fortunately, to design deep learning-based object detectors, there are already many publicly available architectures benchmarked on common datasets, which allow an architecture to be selected based on desired speed and performance characteristics. Instead of training one of these architectures from scratch, we used a transfer learning approach, which takes a pre-trained object detection model as a starting point and transfers the knowledge from its original domain-specific knowledge to another domain [23]. The transfer learning approach is effective as it can take learnt feature detectors from one model that has been exposed to a much larger dataset. These learnt feature detectors may include the ability to detect edges, textures, shapes, and patterns that are applicable in almost any image application. The AI evaluation presented in this research is based on the R-FCN ResNet101 COCO model available from the TensorFlow 1 Detection Model Zoo, as this model showed a suitable compromise between speed and performance [24]. We updated the number of object classes and the label mapping to suit the four helminth egg species available in our training set, but kept the other default parameters, such as data augmentation options and hyperparameters for training, unchanged. The pretrained model and corresponging variables were used as the starting point in the transfer learning process, where these variables are finetuned using the case specific data. Options like the ability to freeze certain layers, which reduces the amount of variables to train and prevents changes to features that may be considered useful, were not explored in this paper.

Our original datasets and several exported model files trained for STH & SCH, including the R-FCN ResNet 101 COCO model presented in this research are made available on Kaggle [https://doi.org/10.34740/KAGGLE/DS/1896823]. The original code using TensorFlow 1 is not supported on Kaggle due to version compatibility, however a working solution in TensorFlow 2 that can extract data, train object detection models and evaluate both TensorFlow 1 and TensorFlow 2 models is made available.

While the provided Kaggle notebooks are working solutions, the free cloud-based graphics processing unit (GPU) available on Kaggle is not sufficient to optimally train the available state-of-the-art object models with the provided data. During our research, the training of the AI model was performed on an NVIDIA GeForce RTX 2070 GPU, while the evaluation of the unseen test set was performed on a Jetson AGX Xavier Developer Kit. The AGX represents a potential fieldable computer for the use of AI models without an internet connection to a cloud server where commercial solutions typically process images.

The performance of the AI model on the unseen test was obtained by comparing the manually verified ground truth to that predicted by the AI model in a confusion matrix. The recall (a perfect recall score has no false negatives), precision (a perfect precision score has no false positives), and F1-score (harmonic mean of recall and precision) were calculated. Evaluation measures involving true negatives in object detection are typically not used due to the sheer number of detections made for every FOV image (the model may generate thousands of regions of interest for evaluation on every FOV image). Every evaluated region within the FOV image that is correctly classified as a negative (e.g. some debris) would increase the true negative count, significantly outnumbering the true positives, false positives, and false negatives for a given FOV. Clinical sensitivity and specificity for the AI model on KK stool thick smears were not evaluated in this research for several reasons: (i) many scans did not cover the complete KK stool thick smear area, therefore it was not possible to count all eggs per slide, (ii) the tool lacked the functionality to distinguish duplicate eggs in overlapping FOV images, and (iii) egg counts using microscope visual examination for the scanned slides were not recorded.

## Results

In this section, we present the results from evaluating the WSI scanner in the field, the specifics of the image database collected from field trials and the performance of the AI model on the unseen test set.

### Performance of the WSI scanner

**Fig 3:**
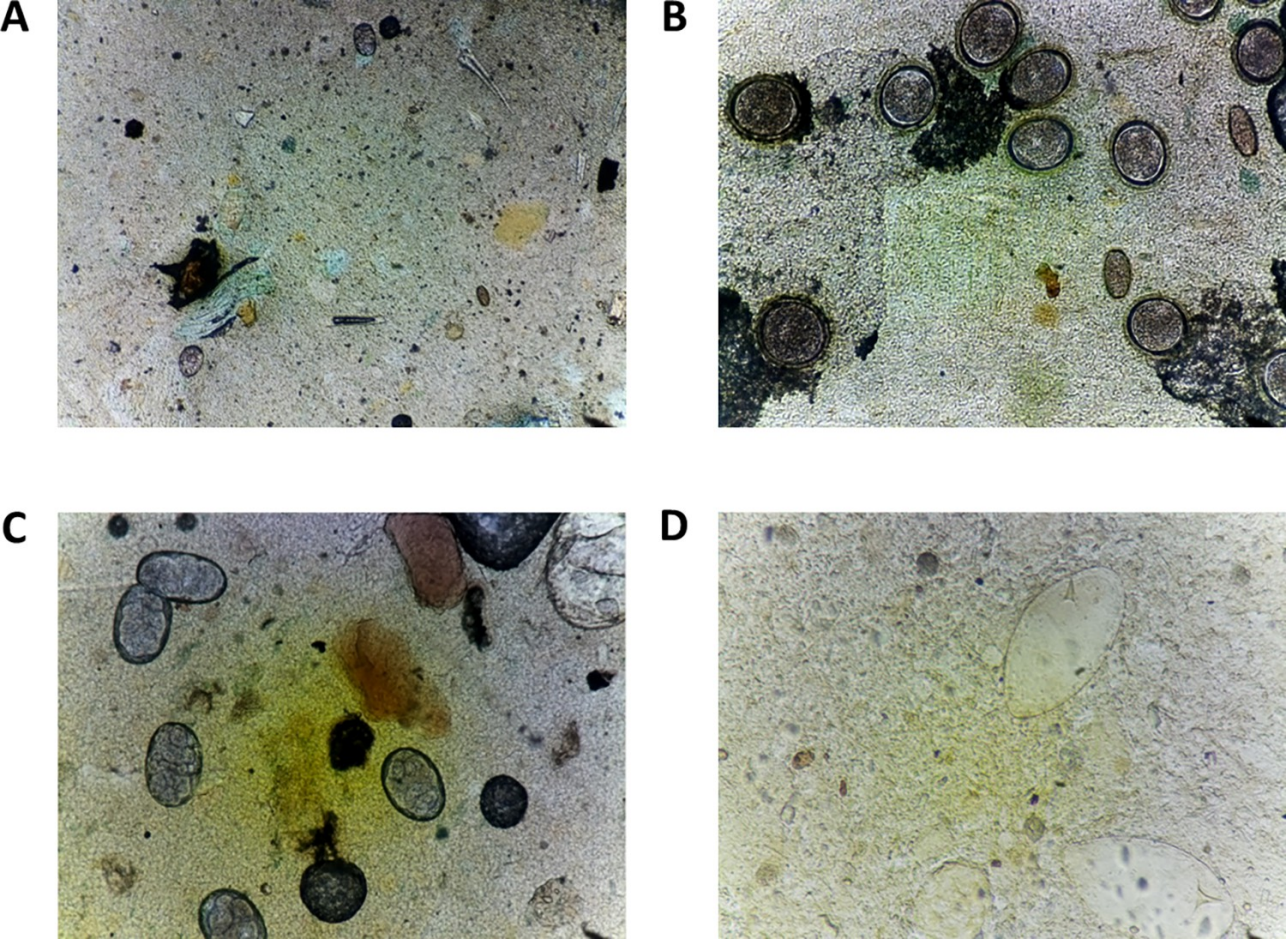
Example images collected using the WSI scanner. (A) Image at 4x objective with two hookworm eggs and one *Trichuris* egg. (B) Image at 10x objective with multiple *Ascaris lumbricoides* and *Trichuris trichiura* eggs. (C) Image at 10x objective with five hookworm eggs. (D) Image at 10x objective with two *Schistosoma mansoni* eggs.

The low-cost optical components (configured with 10x 0.25NA objective), diffused white LED, and 8MP camera were found to achieve sufficient spatial resolution for analysis and manual annotation of the helminth eggs. While the helminth eggs were observable with the 4x objective lens (**Fig 3A**), verification was preferred with the 10x objective (**Fig 3B–3D**).

The trials embedded with the WSI scanner resulted in the scanning of over 300 KK stool thick smears (**Table 1**). The evaluation of several scanning methods revealed that capturing focus stacks for each FOV was the most robust procedure (**S1 Fig**). The scanning time for a complete smear was around 90 minutes and therefore complete stool thick smears were never imaged. It took around 30 minutes to scan a range of 1,000 FOV in one slide. In the first three trials (Cambodia, Kenya, and Tanzania), images were collected as focus stack scans of between 500 to 1,000 FOVs per slide (being a subsection of the complete sample area). In Ethiopia, we scanned slides using a new scanning method, continuously capturing images on a single focus plane scan. The scan method allowed complete sample areas to be scanned in under 20 minutes resulting in the collection of many unique slides scanned and unique FOV images captured. However, using a single focus plane a large portion of the collected images was not sufficiently in focus for analysis and hence this collection was excluded for the AI training.

**Table 1:** Summary of the Kato-Katz slide images collected with the prototype WSI.

Study location	Scanned slides	Collected images (images/FOV)	Images with focus verified^2^	Verified parasitic elements^3^ from focused images in focus stacks				Totals
				*Ascaris*	*Trichuris*	Hookworm	*Schistosoma*	
**Tanzania**	54	123,409 (4–8/FOV)	4,928	8,600	4,083	14	3^4^	12,700
**Cambodia**	76	292,344 (4–8/FOV)	1,129	0	0	3,609	0	3,609
**Kenya**	>30^1^	103,462 (4–8/FOV)	1,723	0	0	0	681	681
**Ethiopia**	142	866,971 (1/FOV)	-	-	-	-	-	-
**Totals:**	272	1,386,186	7,780	8,600	4,083	3,623	684	16,990

^1^slide identifiers were not recorded in the database

^2^ “Images with Focus Verified”: column shows the number of images that have been manually verified, but these are not the exhaustive set of focused images available.

^3^ “Verified Parasitic elements” have been extracted from the focused images by AI and manually reviewed.

^4^ At least one of the *Schistosoma* eggs recorded was found to be wrongly verified. No *Schistosoma* eggs were expected from the study in Tanzania.

FOV = field of view.

### Build-up of the image database

In total, over 1.3 million FOV images from KK stool thick smears were captured of which 7,780 unique and focused FOV images were selected. From these, 16,990 images of helminth eggs were extracted and reviewed across three STH species (*Ascaris lumbricoides*: 8,600, *Trichuris trichiura*: 4,083, hookworms: 3,623, and SCH: 684; **Table 1**). The 7,780 focused FOV images with the labelled egg annotations are available for research purposes [https://doi.org/10.34740/KAGGLE/DS/1896823]. The total number of eggs and their distribution between the training, validation and test sets are summarised in **Table 2**. FOV images containing multiple eggs of the same or different helminth species were root cause to an unprecise split into the targeted percentages.

**Table 2:** Number of parasite eggs used in the training sets.

	Distribution of eggs by type and data set after random shuffling (distribution of eggs by data set for the given egg type, distribution of eggs by type for the given data set)				
Data sets	*Ascaris*	*Trichuris*	Hookworm	*Schistosoma*	Totals
**Train set (70% target)**	6,119 (71.2%, 51.2%)	2,839 (69.5%, 23.8%)	2,514 (69.4%, 21.0%)	477 (69.7%, 4.0%)	11,949 (70.33%, 100%)
**Validate set (20% target)**	1,648 (19.2%, 48.9%)	859 (21.0%, 25.5%)	721 (19.9%, 21.4%)	142 (20.8%, 4.2%)	3,370 (19.8%, 100%)
**Test set (10% target)**	833 (9.7%, 49.9%)	385 (9.4%, 23.0%)	388 (10.7%, 23.2%)	65 (9.5%, 3.9%)	1,671 (9.8%, 100%)
**Totals**	8,600 (100%, 50.6%)	4,083 (100%, 24.0%)	3,623 (100%, 21.3%)	684 (100%, 4.0%)	16,990 (100%, 100%)

### AI training and evaluation

Having trained the AI model with the training set (parameter tuning using transfer learning) and validate set (hyper parameter tuning) (**Fig 1**), the performance was finally evaluated on the unseen test set. The final test set contained 1,671 manually verified STH and SCH eggs (derived from 752 unseen and unique FOV images). The results are shown in the confusion matrix of **Table 3**, comparing the manually verified ground truth with those predicted by the AI model. The selected AI model correctly identified 1,605 of the 1,671 (96.1%) STH and SCH eggs while 66/1,671 (3.9%) eggs from the ground truth were missed by the AI model (false negatives). A total of 87 background stool artefacts from the FOV images were wrongly classified by the AI model as eggs (false positives). No misclassifications between any of the helminth egg species were observed.

**Table 3:** Confusion matrix and performance measures for STH and SCH AI model.

		*AI predictions and performance*				False negatives (missed eggs)		
		*Ascaris*	*Trichuris*	Hookworm	*Schistosoma*			
*Verified ground truth*	*Ascaris*	799	0	0	0	34		
	*Trichuris*	0	371	0	0	14		
	Hookworm	0	0	379	0	9		
	*Schistosoma*	0	0	0	56	9		
False positives (background artefacts)		39	23	20	5	-	Weighted average	Standard deviation
Recall (%)		95.9	96.4	97.7	86.2		96.1	2.1
Precision (%)		95.4	94.2	95.0	91.8		94.9	0.8
F1-Score (%)		95.6	95.3	96.3	88.9		95.4	1.4

From the confusion matrix’s true positives, false negatives, and false positives, the recall, precision and F1-score for each egg species were calculated. As discussed in the method section, true negatives are not helpful due to the sheer number of regions analysed by the AI model within the FOV images. Overall, our results demonstrate a high weighted average precision (94.9 ± 0.8%) and a high weighted average recall (96.1 ± 2.1%) across all egg species on the unseen test set.

Evaluation of the AI model with the unseen test set was carried out with the AGX computer to demonstrate the possibility of the using the model in offline and remote settings. The model achieved an average inference time (the time spent per image to identify and classify all egg features) of 1.58 seconds per image with the trained R-FCN ResNet101 model.

## Discussion

### Proof-of-concept for affordable digital pathology for KK stool thick-smears

The WHO TPP for STH describes a number of test requirements for quantitative detection of analytes specific to STH in all age groups [8]. Laboratory-based tests should be performed in regional or national diagnostic testing laboratories by trained laboratory technicians with less than one week of training, while specific requirements for portability and transport should not exceed those of standard laboratory equipment. Any equipment used for reading the test should be highly portable and battery powered if it needs electricity. The test should be specific (≥ 94%) and have a sensitivity of at least 60% for each STH (*Ascaris*, *Trichuris*, and hookworm), although different sensitivity/specificity combinations are possible. The test should allow for a throughput of at least seven samples per hour and its cost should not exceed USD 3 per test, with maximal equipment cost not exceeding USD 5,000. Starting from this TPP, the designs for AI-DP are very stringent, focusing largely on cost and turn-around time on the hardware, and performance (clinical sensitivity and specificity) on AI.

To explore all the design options for the hardware, we built a first prototype (at under USD 2,000, see **S1 Info**) and used it in real-world settings. The prototype scanner proved useful to evaluate various optic configurations, magnifications, lighting conditions and scanning settings in field settings. The use of low-cost optics and illumination provided sufficient resolution and contrast in images, which significantly simplifies the design and cost for fieldable WSI scanners. This contrasts with the typical use of high-quality objectives and more complex illumination with condensers and multiple adjustable iris diaphragms (as required with Köhler illumination) used in standard laboratory microscopes. With this prototype device, we demonstrated that KK stool thick smear slide scanning in field settings is technically possible.

### Lesson learned from the field

Our experience with using the WSI scanner in STH and SCH field settings in 4 different endemic countries highlighted numerous challenges, including (i) the portability of the device, (ii) the usability of the prototype scanner, (iii) the environmental conditions to which the WSI-scanner was exposed, and (iv) the variability in the KK stool thick smears.

#### The portability of the device

The device was packed into a transport case (**S2 Fig**), which was robust for travelling, but it would not be practical for more extensive surveys requiring multiple units. The setup and operation of the device required mains power which is not always available in field settings. Furthermore, all field settings, including laboratories, were subject to frequent power interruptions during a typical day. Although the outages may last only seconds or minutes, the power interruptions also interrupted the scanning process and resulted in the loss of data for the current scan. While some other WSI approaches require cloud-based operations [15–17], access to the internet was very limited and unreliable in the remote endemic areas where our prototype systems were evaluated. Potential solutions for this first challenge would be to reduce the size of the WSI scanner to enable more scanners per equipment case and to integrate or include an uninterruptible power supply (UPS) with battery backup with the system. Additionally, the solution must operate completely independent of a connection to the internet. Notably, with the current state of internet infrastructure, mHealth/telehealth solutions relying on the internet for image transfer or image analysis by cloud-based AI models cannot be relied on. The use of fieldable embedded computers like the Nvidia AGX enables powerful AI models to be deployed in the field and generate results in a timely manner without dependence on internet infrastructure.

#### The usability of the prototype scanner

The device included a small touch screen for initialising and monitoring of the scanning process. While the screen and touch interface were beneficial for feedback, the process to manually enter slide details, set-up the sample boundary and review focus points was time-consuming and complex for untrained users. Potential solutions for this second challenge would be to allow automated barcode/QR code reading to capture slide details and to automatically set the scanning area with detection of the smear boundary, as typically done by commercial WSI scanners.

#### The environmental conditions to which the WSI-scanner was exposed

During the field studies, the WSI scanner was exposed to a variety of environmental conditions, including high temperatures, high humidity, and fine dust. Due to the flexibility and open nature of the prototype, regular maintenance with cleaning and lubrication was required for the XYZ-axis automation to ensure smooth operation. Potential solutions for this challenge would be to shield hardware from environmental conditions.

#### The variability in the KK stool thick smears

Slide preparation is standardised as per WHO protocol, from which we wanted to deviate as little as possible. Nevertheless, important variations existed in shape, size, thickness, uniformity, appearance, consistency of the stool thick smears, and the visibility of eggs. Due to the challenges faced by the variations in the stool thick smears, the focus stacking scan method proved most reliable. However, as also noted by Bian et al. [25], the focus stack scanning method is the most time consuming scan method, generates the most data with many images (between 0.5 MB to 1 MB per image without compression) and requires additional processing time.

Due to 90-minute scan time of the prototype WSI scanner, and the limited time window of 30 minutes in which smears should be scanned to prevent the loss of hookworm eggs, the scanning of complete slides was de-prioritised. Instead, collecting images during the 30-minute time window for as many slides as possible became the main objective. This typically resulted in the order of 500 to 1,000 unique FOVs per slide. Since the thickness of the KK stool thick smear determines the number of images per stack, the scanning device requires a precise motorised stepping system on the Z-axis. As demonstrated in the field trial in Ethiopia, scanning slides using a single linearly interpolated focus layer resulted in out-of-focus images from which it was difficult to manually extract useful images for training purposes, but also for egg detection purposes.

Potential solutions for this challenge would be to significantly reduce scanning time such that entire samples can be scanned within the time window where hookworm eggs are still present. This could be realised by implementing adaptations to the magnification, the size of the FOV, and/or using camera sensors with large frame size and higher framerate, and increased computing power [25]. Other approaches might include deviations from the standardised sample preparation and processing, as for example the use of iodine staining solution with 40% acrylamide gel preparations or other fixation and sample preparation technologies [15]. However, these adaptations are rather tools for proof-of-principle experiments in laboratories settings, and possibly not feasible for remote endemic areas. Technologies that lack the stepwise Z-axis image stacking procedure will most likely not be useful for KK stool thick smears. Single focus layer automated scanning is in general the principle used in handheld and mobile phone microscopy [16,18,26,27].

In conclusion, it became clear that this prototype WSI scanner was useful to build an image library, but significant design changes are needed to transform the current design into a truly feasible and fieldable WSI scanner. To comply with the WHO TPP specifications for hardware, we envision several rounds of prototyping and field evaluations as future work.

### Collection of STH and SCH images

Our curated database extends previous literature by collecting focus stacks of STH and SCH eggs from over 300 freshly prepared human KK stool thick smears under typical field and study settings. The collection of over 1.3 million images containing 16,990 verified egg annotations across four helminth species was collected from four geographic regions with our prototype WSI scanner, which provided added variability in the stool appearance and provided valuable and practical lessons in how AI-DP solutions can fit into field settings. As a comparator to another KK image collection, the Kankanet approach used a dataset of 2,078 images and the unseen dataset was based on 185 egg images [18], or a test set (15%) that consisted of 136 *Trichuris* and 647 *Ascaris* egg images [17]. Other AI models that were not trained and evaluated with images collected from KK stool thick smears are likely not transferable to KK images and are likely to experience a significant reduction in precision and recall due to the additional egg-like artefacts and debris present in KK stool thick smears. FecalNet [28] for example, has been evaluated on 5,279 hookworm eggs (89.95% precision, 93.88% recall), 19,708 *Ascaris* eggs (96.90% precision, 91.21% recall), and 2,178 *Trichuris* eggs (88.61% precision, 94.37% recall), however the samples were not prepared with the KK technique.

A limitation of the current database is the uneven distribution of eggs per class, with approximately 50% of eggs for each data set belonging to *Ascaris*. The annotated eggs are also unevenly distributed between stool samples of varying infection intensity. Most eggs in the dataset come from few slides of high infection intensity; thus, the model may contain bias toward stool samples of a particular appearance. Future work will focus on expanding the database and ensuring more even distribution of eggs from unique stool samples of varying infection intensity, and from different geographical regions.

### Performance of the AI-DP approach

Concerning the WHO TPP requirements for test performance, a clinical sensitivity of ≥ 60% and a clinical specificity of ≥ 94% is needed. The KK stool thick smear technique is generally seen as a method with sensitivity around 70%, but the specificity of the determinations is near perfect ≥ 99%–as the latter depends on the operator making the decisions [29,30]. The goal of implementing AI on KK should therefore aim to increase the clinical sensitivity, while maintaining the almost perfect clinical specificity. In this research, it was not possible to determine the clinical sensitivity or specificity of the system at a sample level, for reasons discussed in the Methods section. Instead, we provide the analytical performance (recall and precision) for an AI model to identify and classify STH and SCH eggs from FOV images within a sample. Our results of the AI model demonstrate a high average weighted precision (94.9 ± 0.8%) and weighted average recall (96.1 ± 2.1%) across all species. The lower precision (91.8%) and lower recall (86.2%) for SCH can largely be attributed to the limited size of the training data collected. The performance of our AI model for STH and SCH is comparable to or better than previously published AI based approaches [15,17,18,28,31–35].

Our results suggest that the required clinical sensitivity and specificity are not out-of-reach for an AI-based system. However, it is important to recognise that the clinical sensitivity and specificity of the system will depend on other system parameters and algorithms such as (i) the ability to capture all FOVs within the sample, (ii) the ability to ensure each FOV contains an in-focus image, and (iii) the ability to distinguish between eggs in over lapping FOVs. Further increasing the clinical sensitivity of the model can be achieved by (i) annotating additional eggs missed in ground truth training sets: having missed egg annotations in the training sets negatively impacts the final model, (ii) building up the data base to remove bias from certain regions and specific stool samples: by having a more diverse training set, the generalisability of the model improves and k-fold cross validation can be used to reduce bias while training and better evaluate the performance of the AI model on unseen data, (iii) improving the scanning time such that whole smears are scanned, (iv) using a KK template with a larger stool volume, and (v) optimising the confidence threshold in deciding whether detections are positive or false. For the latter, the model can be tuned for the desired performance by analysing the receiver operator curve (ROC) for precision and recall with varying confidence cut off thresholds.

For the clinical specificity of an AI-DP system, many additional design steps can be considered. For example, in the manual procedure, the high specificity is obtained by decisions made by a trained microscopist observing the eggs visually. The AI performance on our test set is missing this manual curation by a trained microscopist. A hybrid AI-specialist approach, in which AI is preselecting the features from the KK stool thick smears, and an operator is deciding on the final determination might easily push an AI-DP system’s clinical specificity to the ≥ 99% requirement. This could be reached by developing a specific GUI for feature determination. However, this is easier said than done and can only be a successful approach if the amount of false positive features remains manageable. From evaluation of our AI model, we demonstrate a 5.2% false positivity rate (87 false positives for 1,671 true positives). As KK stool thick smears are composed of a complex and diverse matrix containing endless variations of egg-like features, it might be necessary to extend the data collection, the egg annotation activities, and the AI retraining on ever increasing gold truth egg collection. An optimal focus of the presented image is another requirement for reliable AI function. The AI model was trained on in-focus FOV images. In our experience, the egg-like features seen in out-of-focus images will negatively influence the false positivity rate. When applying the current AI model to the out-of-focus images of KK stool thick smears scanned in Ethiopia, we retrieved countless egg-like features, in which the true positives were deeply buried in an overwhelming number of false positives. Hence, clinical performance will be dependent on proper scanning methods, a perfect control of in focus-images for AI interpretation, and hybrid approaches in which the operator makes the final decision (manually removing the false positives).

### Assessing STH specific intensity of infections in case of co-infections

The results indicate that STH-specific egg counts can be reported for each parasite separately. This solution shows great promise in its ability to multiplex for co-infection sites with very little expected dependency on infection intensity or prior knowledge on the prevalence in the study area. While the egg counting process of a microscopist is not always independent (once an egg is detected in FOV, one will be more attentive to find more), decision-making of our AI model is not affected by finding eggs in a previous FOV, nor by whether other samples in the region contain eggs. While this research has focused on the analytical performance of the AI model on individual images collected from high infection intensity samples, further evaluation of the system’s diagnostic performance on low infection, medium and high infection samples is required.

## Conclusion

The development of AI-DP diagnostic tools for NTDs has been limited due to affordability, clinical performance, operability, and usability. In this study, we prototyped an affordable WSI scanner capable of scanning KK stool thick smears. While we are yet to demonstrate clinical sensitivity or specificity for an AI based system, our transfer learning approach from existing state-of-the-art object detection models with annotated data from high infection intensity slides, achieved comparable or better analytical performance for recall and precision to extract and classify STH and SCH eggs in comparison to previous literature. Further research and development will be required to adapt the prototype WSI scanner to field settings, demonstrate the AI in end-to-end testing during field use, and evaluate the performance in low, moderate and heavy infection intensity settings. However, we are hopeful that future affordable and fieldable AI-DP scanners might yet support the ambitious WHO 2030 roadmap for STH and SCH.

## Supporting information

S1 Info Bill of materials for the prototype whole slide image scanner. (PDF)

S1 Fig Scanning procedure used to scan Kato-Katz slides. (TIF)

S2 Fig Equipment case used to transport the prototype whole slide image scanner. (TIF)
