# Supplementary material for: Affordable artificial intelligence-based digital pathology for neglected tropical diseases: A proof-of-concept for the detection of soil-transmitted helminths and Schistosoma mansoni eggs in Kato-Katz stool thick smears
Source: PLoS Negl Trop Dis. 2022 Jun 17;16(6):e0010500. doi: 10.1371/journal.pntd.0010500 (PMC9258839; doi:10.1371/journal.pntd.0010500)
Supplement: S1 Info — (PDF) [file pntd.0010500.s001.pdf]

Bill of Materials for prototype Whole Slide Scanner.

Component	Unit Cost in Euro	Supplier
Lens 10mm focal length (1)	95	Edmund Optics
24V to 5V 5A DC-DC convertor (1)	7	Elecktrokit
24V power supply (1)	26	Elfa
Motor couplings (3)	10	HIWIN
Cross link axis adapter (1)	27	HIWIN
Z motorised axis (1)	271	HIWIN
XY motorised axes (2)	522	HIWIN
RPI Camera V2 8MP (1)	38	Many suppliers
RPI Computer 3 B+ (1)	45	Many suppliers
RPI 7" HDMI Touchscreen (1)	51	Many suppliers
CNC board and preconfigured SD card (1)	36	Protoneer
Cooling fan (1)	6	RS
Aluminium Strut, 40 x 40 mm, 8mm Groove , 2000mm Length (1)	45	RS
Aluminium Strut, 40 x 80 mm, 8mm Groove , 2000mm Length (1)	77	RS
Aluminium Strut fixtures and accessories (Caps, angle brackets, screws, feet)	100	RS
Educational microscope (1)	126	Sagitta
Stepper Motors (3)	27	Stepper Online
Stepper Driver Protectors (3)	18	Watterott
Stepper Motor Drivers (3)	30	Watterott
3D printing material for brackets, led, computer, power supply enclosures	30	Many suppliers
Total (€)	€ 1,587	
