# Supplementary figures and images for: Affordable artificial intelligence-based digital pathology for neglected tropical diseases: A proof-of-concept for the detection of soil-transmitted helminths and Schistosoma mansoni eggs in Kato-Katz stool thick smears
Source: PLoS Negl Trop Dis. 2022 Jun 17;16(6):e0010500. doi: 10.1371/journal.pntd.0010500 (PMC9258839; doi:10.1371/journal.pntd.0010500)

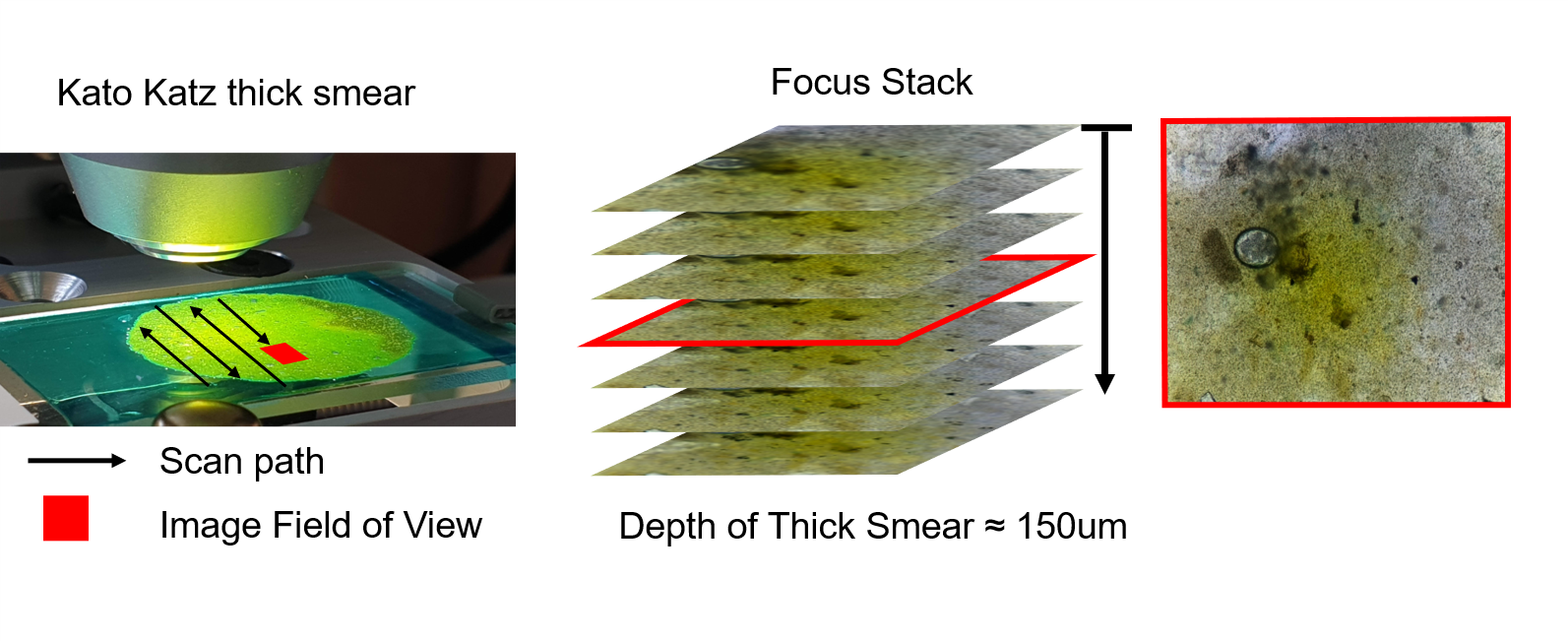

Supplement: S1 Fig — (TIF) [file pntd.0010500.s002.tif]

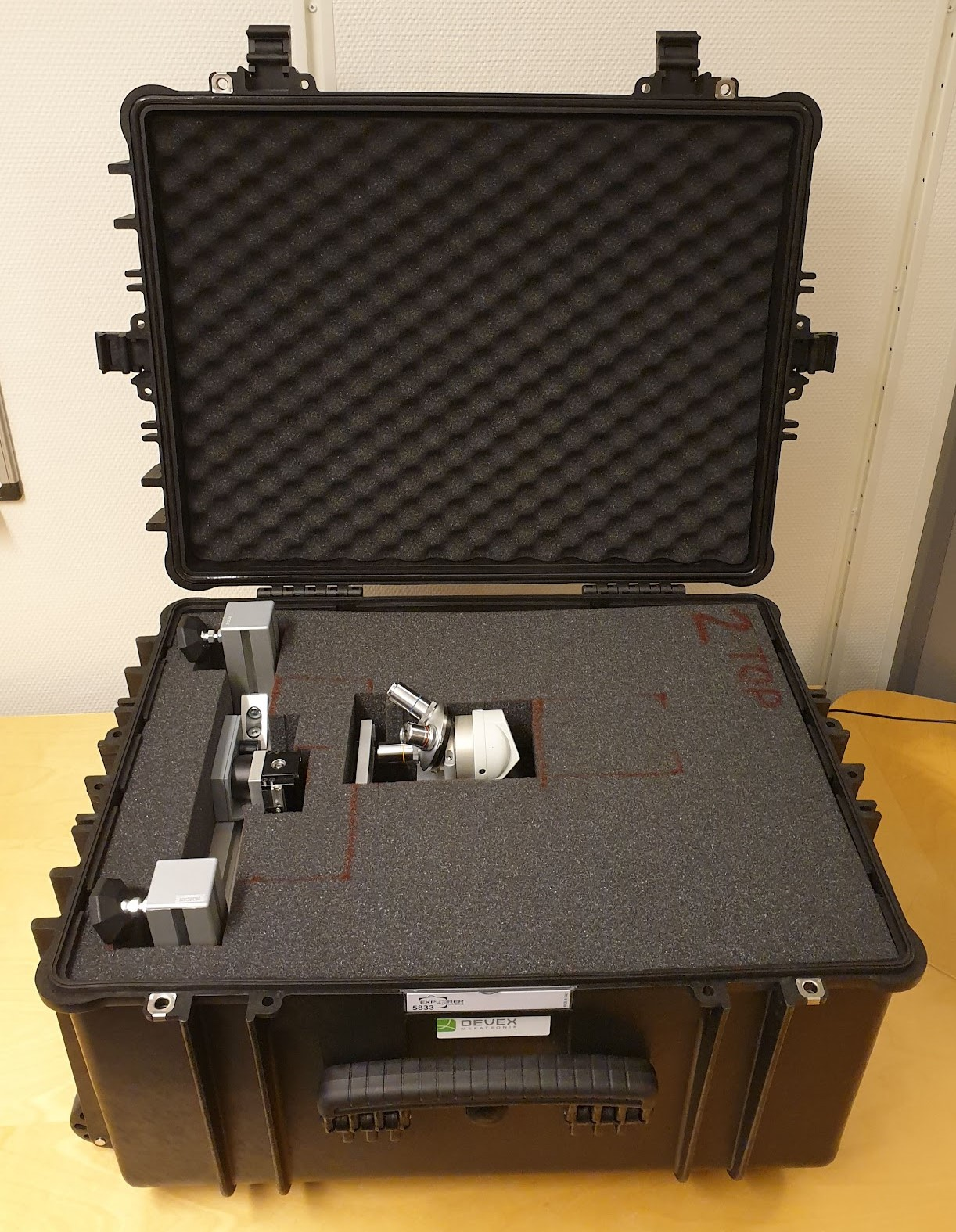

Supplement: S2 Fig — (TIF) [file pntd.0010500.s003.tif]
